# C-MHAD: Continuous Multimodal Human Action Dataset of Simultaneous Video and Inertial Sensing

**DOI:** 10.3390/s20102905

**Published:** 2020-05-20

**Authors:** Haoran Wei, Pranav Chopada, Nasser Kehtarnavaz

**Affiliations:** Department of Electrical and Computer Engineering, University of Texas at Dallas, Richardson, TX 75080, USA; Pranav.Chopada@utdallas.edu (P.C.); kehtar@utdallas.edu (N.K.)

**Keywords:** public domain dataset for multi-modal action recognition, recognition in continuous action streams, fusion of video and inertial sensing for action recognition

## Abstract

Existing public domain multi-modal datasets for human action recognition only include actions of interest that have already been segmented from action streams. These datasets cannot be used to study a more realistic action recognition scenario where actions of interest occur randomly and continuously among actions of non-interest or no actions. It is more challenging to recognize actions of interest in continuous action streams since the starts and ends of these actions are not known and need to be determined in an on-the-fly manner. Furthermore, there exists no public domain multi-modal dataset in which video and inertial data are captured simultaneously for continuous action streams. The main objective of this paper is to describe a dataset that is collected and made publicly available, named Continuous Multimodal Human Action Dataset (C-MHAD), in which video and inertial data stream are captured simultaneously in a continuous way. This dataset is then used in an example recognition technique and the results obtained indicate that the fusion of these two sensing modalities increases the F1 scores compared to using each sensing modality individually.

## 1. Introduction

Many techniques have been developed in the literature for human action or gesture recognition spanning a wider variety of applications, e.g., human–machine interface [1], intelligent surveillance [2,3], healthcare monitoring [4,5] and gaming [6,7]. These techniques normally use a single sensing modality such as video cameras, e.g., [8,9], depth cameras, e.g., [10], or wearable inertial sensors, e.g., [11,12].

Each sensing modality has its own strengths and limitations. For example, video cameras provide rich visual information while being limited in their field of view and sensitive to lighting changes. Depth cameras provide 3D information while their use is often limited to indoors due to their utilization of infrared light. Wearable inertial measurement units (IMUs) or wearable inertial sensors provide acceleration and angular velocity signals irrespective of field of view and lighting changes while being limited in capturing a rich representation of an action performed by several body parts. In other words, no sensing modality can cope with various situations that occur in practice or in realistic scenarios.

Attempts have been made in fusing multiple sensing modalities. It is shown that fusion of video and depth data can generate more robust recognition compared to using only one sensing modality, e.g., [13,14]. There exist vision-based datasets in the literature providing video, depth, skeleton data simultaneously, e.g., PKU-MMD [15], NTU RGB+D [16] and NTU RGB+D 120 [17]. Fusion of the two sensing modalities of depth camera and inertial sensor are also reported in [18,19,20,21,22,23,24] for the purpose of achieving more accurate or robust human action recognition compared to situations when using each sensing modality individually. Furthermore, more recently the two sensing modalities of video camera and inertial sensor have been used simultaneously [25].

Previous works on action or gesture recognition normally assume that actions of interest are already segmented. In [25], it was also assumed that the actions of interest had already been segmented. However, in real-world applications, actions that are of interest appear among actions of non-interest without pauses in between. Recognizing actions of interest in continuous action streams is more challenging since the start and end points of actions of interest are unknown and need to be detected first in real-time as a prerequisite to recognition.

Currently, there exists no public domain dataset in which both video and inertial data are captured simultaneously for continuous action streams. This paper provides a public domain Multimodal Human Action Dataset named C-MHAD with C indicating that actions appear in continuous action streams with no segmentation. The subjects who participated in putting together this dataset were given complete freedom to perform any actions of non-interest as per their choice. This dataset reflects a more realistic scenario when actions are performed in real-world settings and can be used by researchers to examine the effectiveness of their action recognition techniques in a more challenging scenario. The main objective of this paper is to describe a dataset that is collected and made publicly available, in which video and inertial data streams are captured simultaneously in a continuous way. The C-MHAD dataset can be downloaded from this link www.utdallas.edu/~kehtar/C-MHAD.html.

Using a combination of video and inertial sensing for multi-modal continuous action detection and recognition can be utilized in many applications. For example, in gaming to have more reliable interactions, both a video camera and an inertial handheld controller can be used at the same time. Another application example includes a more reliable monitoring of movements of patients in hospital rooms via a video camera and an inertial sensor worn on patients’ wrists. In addition, the combination of video and inertial sensing can be used for assistive living to enable a more reliable monitoring of elderly people’s falls. As a more specific application, sit-to-stand and stand-to-sit transition movements have been used in [26] to assess mobility impairment in multiple sclerosis by using inertial sensors. In addition to inertial sensors, video can be used to increase the reliability of the assessment.

## 2. Existing Public Domain Datasets of Simultaneous Vision and Inertial Sensing

This section gives a brief description of the existing multimodal human action recognition datasets. There are two multimodal datasets that have been widely used. These datasets are the Berkeley Multimodal Human Action Database (MHAD) [27] and the University of Texas at Dallas Multimodal Human Action Dataset (UTD-MHAD) [28]. The UTD-MHAD dataset also includes an augmented dataset [22,24] in which depth images and inertial signals are provided simultaneously for continuous action streams. There exists no public domain dataset in which video images and inertial signals are provided simultaneously for continuous action streams.

The MHAD dataset contains 11 actions performed by 12 subjects (five female and seven males). Each subject performed five repetitions of each action, yielding a total of 660 action segments. Each action was simultaneously captured in five different ways: by an optical motion capture system, by a stereo vision camera system, by Kinect depth cameras, by three-axis accelerometers, and microphones.

The UTD-MHAD dataset contains 27 actions performed by eight subjects (four females and four males). Each subject performed four repetitions of each action, yielding a total of 864 action segments. Each action was simultaneously captured by a Kinect camera and a wearable inertial sensor. The Kinect data consists of depth images and the inertial data consists of three-axis accelerations and three-axis angular velocities.

The UTD-MHAD dataset was augmented by another dataset reported in [22] for continuous action streams. The subjects in this augmented dataset were asked to wear a wearable inertial sensor on their right wrist. In this dataset, the depth images and inertial signals associated with the five actions or gestures of interest in the smart TV application were collected. The five actions or gestures of interest were performed randomly among various actions or gestures of non-interest in a continuous manner by 12 subjects (three females and nine males). Each subject performed an action of interest 10 times. For testing, the subjects performed the five actions of the smart TV application among arbitrary actions of non-interest in a continuous and random manner five times, thus generating a total of 60 continuous action streams. A typical duration of a continuous action stream in this dataset is about 92 s.

Another augmented dataset to the UTD-MHAD dataset is reported in [24] where the actions of interest consisted of seven transition movements between the body states of sitting, standing, lying down, and falling down. The depth images and inertial signals for these actions were captured by a Kinect depth camera and a wearable inertial sensor simultaneously. For testing, five continuous action streams were collected for five subjects for a total of 25 continuous action streams. Each continuous action stream contained the seven transition actions performed randomly among arbitrary actions of non-interest such as stretching, reading a book, drinking water, eating, combing, etc.

The first and second datasets noted above incorporate only segmented actions, while the third and fourth datasets noted above incorporate only inertial and depth data. In what follows, a new dataset that we have collected in which video images and inertial signals are captured simultaneously is reported. This dataset is collected for two sets of actions of interest or applications consisting of smart TV gestures and transition movements.

## 3. Continuous Multimodal Human Action Dataset (C-MHAD)

**Figure 1:**
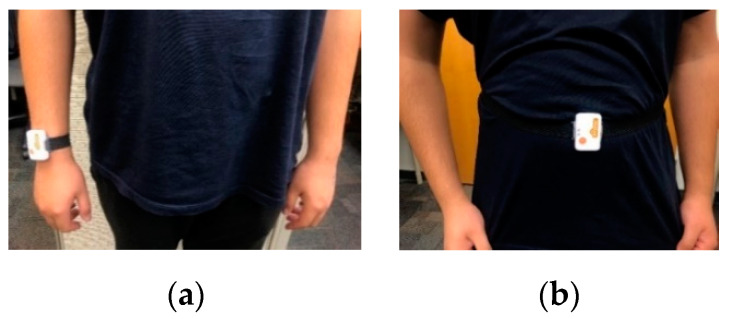
Placement of the wearable inertial sensor: (**a**) right wrist, or (**b**) middle waist.

Considering the unavailability of a public domain continuous multimodal human action dataset where inertial signals and video images are captured simultaneously, a dataset named C-MHAD is collected in this work for two sets of actions of interest corresponding to smart TV gestures and transition movements. The inertial signals in this dataset consists of three-axis acceleration signals and three-axis angular velocity signals. These signals are captured by the commercially available Shimmer3 wearable inertial sensor [29] at a frequency of 50 Hz on a laptop via a Bluetooth link. During data collection, a time stamp was applied to each collected data frame. The videos were captured by the laptop videocam at a rate of 15 image frames per second with a resolution of 640 × 480 pixels. During data capture, the image frames and the inertial signals were synchronized as follows. Along with the acceleration and angular velocity signals, a time stamp was provided by the inertial sensor. The clock function of the video capture code was also used to record the time which was then matched to the inertial sensor time stamp. The captured inertial signals were written to an Excel file, and the video frames were saved in an .avi file. For the smart TV gestures application, the inertial sensor was worn on the subject’s right wrist, as shown in Figure 1a. For the transition movements application, the inertial sensor was worn on the subject’s waist, as shown in Figure 1b.

### 3.1. Smart TV Gesture Application

**Figure 2:**
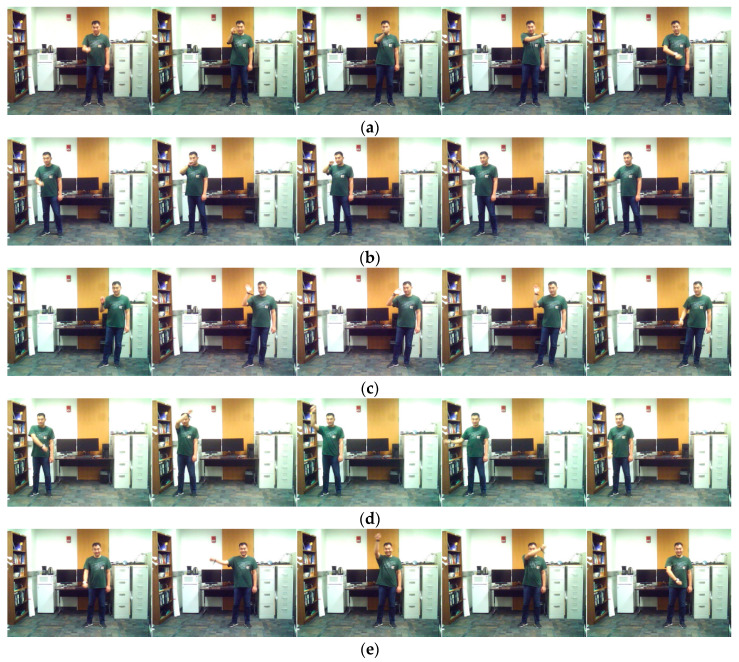
Sample representative image frames for the smart TV gestures application in the C-MHAD dataset: (**a**) swipe to left, (**b**) swipe to right, (**c**) right hand wave, (**d**) clockwise circle, and (**e**) counter-clockwise circle.

**Figure 3:**
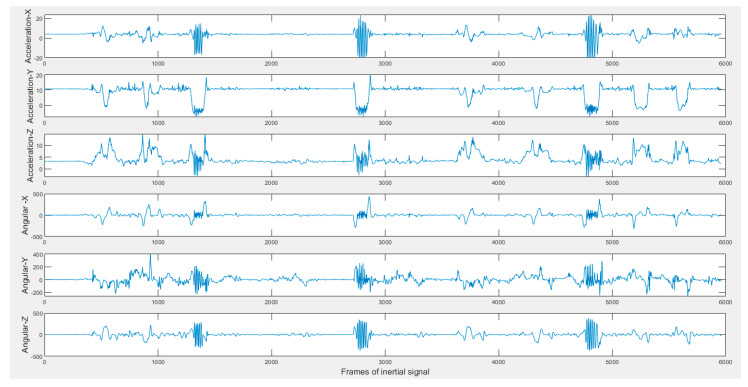
Sample inertial signals in the C-MHAD dataset (top three acceleration and bottom three angular velocity) of a 2-min continuous action streams containing the smart TV gestures or actions.

The continuous dataset for this application contains five actions of interest of the smart TV gestures application performed by 12 subjects (ten males and two females). Ten continuous streams of video and inertial data, each lasting for 2 min, were captured for each subject. The acceleration and angular velocity signals of the Shimmer inertial sensor were streamed in real-time to a laptop via a Bluetooth link. At the same time, the videocam of the same laptop was used to capture video clips with the subject. The subjects moved freely in the field of view of the camera. All the gestures performed by the subjects were done by the arm wearing the inertial sensor. The five actions of interest in the smart TV application that were performed by the subjects are swipe left, swipe right, wave, draw circle clockwise, and draw circle counterclockwise. These gestures or actions are listed in Table 1. Figure 2 shows representative image frames for each action or gesture of interest. Figure 3 shows the six inertial signals (three accelerations and three angular velocities) for a sample 2-min action stream. The subjects performed the actions or gestures of interest in random order during an action stream while arbitrarily performing their own actions of non-interest in-between the actions or gestures of interest.

### 3.2. Transition Movements Application

**Figure 4:**
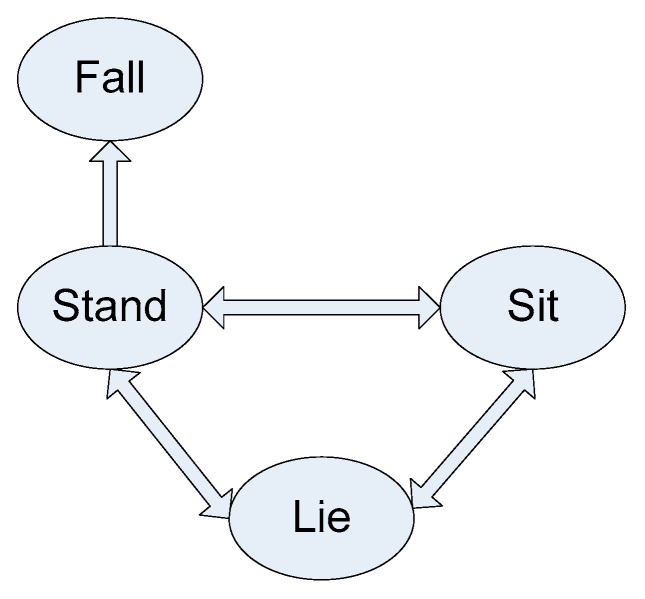
Transition movements actions of interest in the C-MHAD dataset.

**Figure 5:**
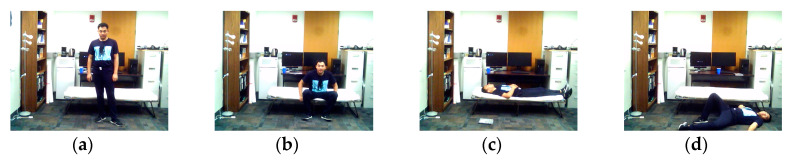
Positions associated with the transition movements application in the C-MHAD dataset: (**a**) stand, (**b**) sit, (**c**) lie, (**d**) fall.

**Figure 6:**
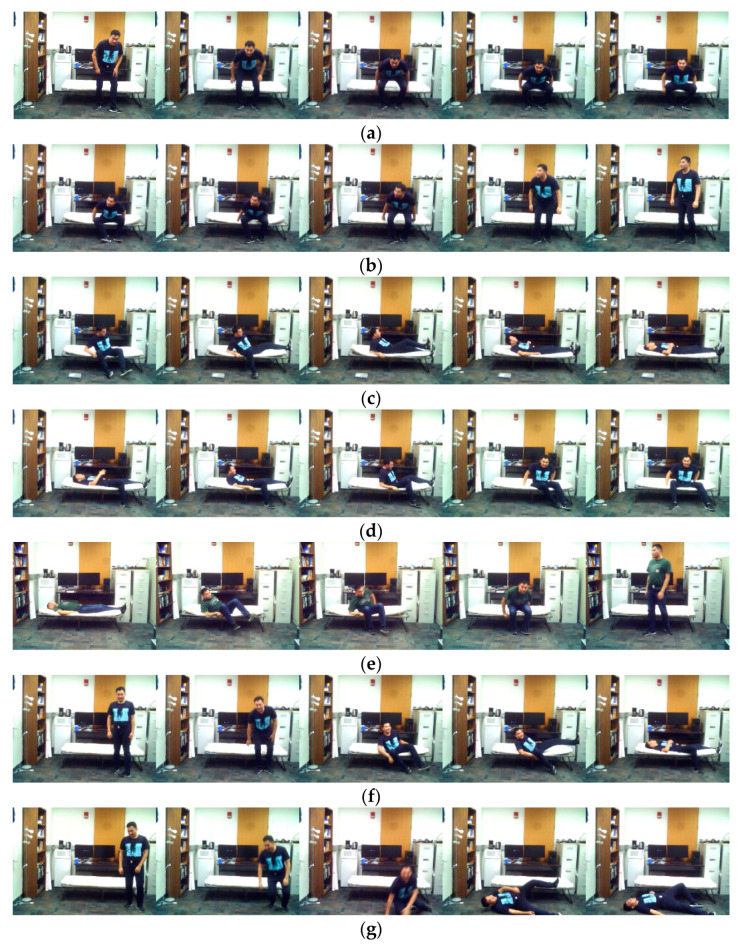
Sample representative image frames for the transition movements application in the C-MHAD dataset: (**a**) stand-to-sit, (**b**) sit-to-stand, (**c**) sit-to-lie, (**d**) lie-to-sit, (**e**) lie-to-stand, (**f**) stand-to-lie, (**g**) stand-to-fall.

**Figure 7:**
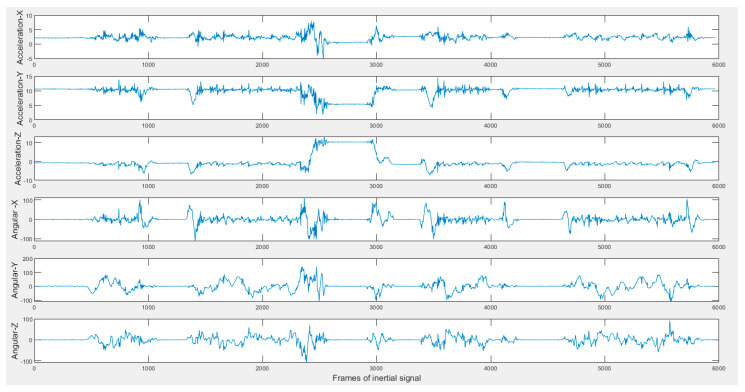
Sample inertial signals in the C-MHAD dataset (top three acceleration and bottom three angular velocity) of 2-min continuous action streams containing the transition movements actions.

**Figure 8:**
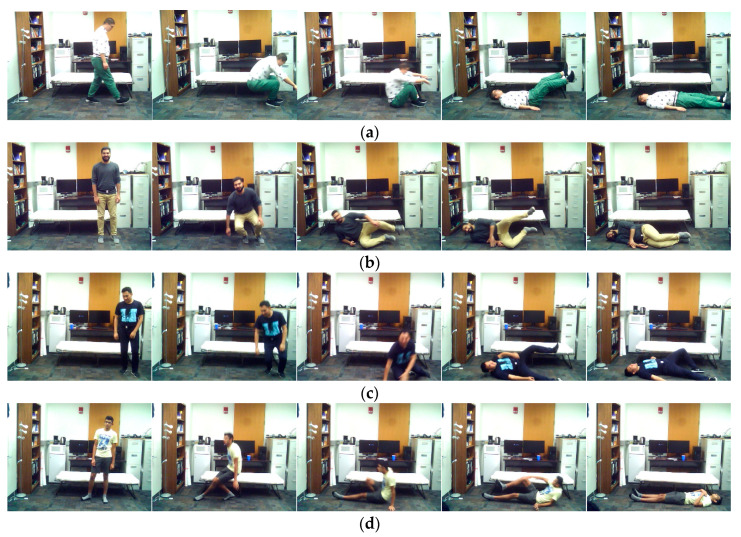
Representative images of action stand-to-fall by (**a**) subject 1, (**b**) subject 3, (**c**) subject 11, and (**d**) subject 12.

The continuous dataset for this application consists of seven actions of interest reflecting transition movements performed by the same 12 subjects (10 males and two females) who performed the smart TV gestures. As depicted in Figure 4, these actions of interest consist of stand-to-sit, sit-to-stand, sit-to-lie, lie-to-sit, lie-to-stand, stand-to-lie, and stand-to-fall. These transition movements take place between four positions of stand, sit, sleep, and fall as illustrated in Figure 5. Figure 6 shows representative image frames for each action of interest. Ten continuous streams of video and inertial data, each lasting for 2 min, were captured for each subject. This time the Shimmer inertial sensor was worn around the waist. The subjects performed the transitions movements in random order during an action stream while arbitrarily performing their own actions of non-interest in-between the actions of interest. A bed was used for the lying down transition movements while the falling down was done from the standing position to the floor. Figure 7 shows the six inertial signals (three accelerations and three angular velocities) for a sample 2-min action stream of the transition movements application. Figure 8 shows representative images of the action stand-to-fall performed by four subjects (1, 3, 11, 12) illustrating that the actions are not performed in exactly the same manner by each subject.

### 3.3. Data Labeling

In total, 240 video clips (120 for the smart TV gestures application and 120 for the transition movements application) were collected from the 12 subjects. Table 2 lists the attributes of the C-MHAD dataset. For accurate recording of the data, the Avidemux video editing software [30] was used with a time extension to see all the individual frames and record the time of the actions of interest in 10-ms increments. Note that the actions of interest are manually labelled in the C-MHAD dataset. In other words, for every action of interest in the action streams, the start and end time were identified by visual inspection of all individual image frames. Although this was a time-consuming process, it provides an accurate label for the start and end time of an action of interest.

## 4. Utilization of C-MHAD in a Sample Recognition Technique

In this section, an example utilization of the C-MHAD dataset is covered by applying a simplified version of a previously developed recognition technique to it. An overview of the recognition technique used is stated next. Readers are referred to [31] for the details of the technique.

**Figure 9:**
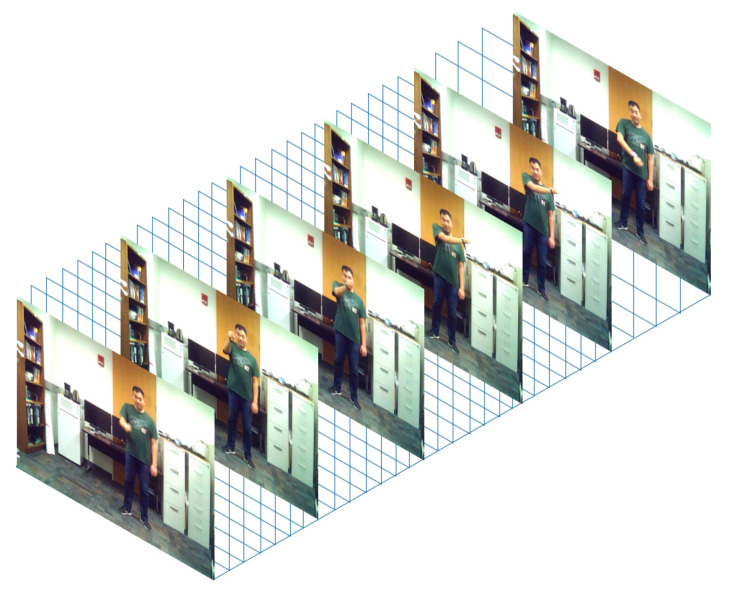
Example image volume as input to the 3D convolution neural network.

For the video data, the actions of interest in each video clip were first converted to 3D image volumes consisting of 45 image frames lasting 3 s in duration. This number was chosen since it included more than 95% of the start and end times of actions of interest. The 3D image volumes were then fed into a 3D convolutional neural network (CNN) whose architecture is described in [31]. To gain computational efficiency, each action image volume had the same spatial and temporal size of 320 × 240 × 45. An example 3D image volume formed in this manner is shown in Figure 9.

**Figure 10:**
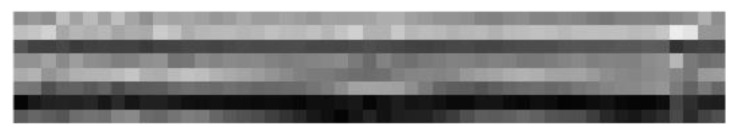
Example inertial signals image as input to the 2D convolution neural network.

For the inertial data, eight-column images were formed by concatenating the three-axis acceleration signals, the three-axis angular velocity signals, the overall acceleration signal, and the overall angular velocity signal. These eight-column images were normalized along each column. The time span of the inertial signal images was also 3 s corresponding to 150 samples provided by the inertial sensor. To make the inertial sampling rate compatible with the video sampling rate and reduce the computation complexity, a median filter of size three was used and only one frame from every three consecutive frames was considered making the signal images of size 8 × 50. An example inertial signal image formed in this manner is shown in Figure 10. These inertial signal images were then fed into a 2D convolutional neural network whose architecture is described in [31].

For fusing the two sensing modalities of video and inertial, a sliding window is used along each sensing modality path to perform action segmentation. A 3D CNN is used for the video data and a 2D CNN is used for the inertial data. The outputs of these models are then concatenated and fed into a fully connected classification layer.

The experiments conducted were conducted for both the smart TV gestures and transition movements applications. For each subject, the continuous action streams numbered 1 through 9 were used for training and the continuous action stream numbered 10 was used for testing and the outcomes were averaged. The recognition process was conducted in two stages. First, actions of interest were separated from actions of non-interest (detection stage). Then, the detected actions of interest were identified (recognition stage). There were errors associated with both the detection stage and the recognition stage. Detection errors involved false positives and false negatives corresponding to actions of non-interest detected as actions of interest and missed detected actions of interest, respectively. Recognition errors involved the confusion matrix errors associated with each action of interest. Table 3 lists the F1 scores of the detection stage for the two applications and for the three sensing modalities of video sensing modality only, inertial sensing modality only, and fusion of video and inertial sensing modalities. Table 4 lists the accuracy of the recognition stage for each action of interest for the two applications and for the three sensing modalities. Table 5 lists the F1 scores when the detection and recognition errors are combined. As can be seen from these tables, the fusion of the two sensing modalities led to higher F1 scores and accuracies compared to when each sensing modality was used individually.

## 5. Conclusions

This paper has made a continuous multimodal human action dataset named C-MHAD available for public use to conduct action recognition via the fusion or simultaneous utilization of inertial and video data. This dataset includes three-axis acceleration and three-axis angular velocity signals from a wearable inertial sensor and video clips from a camera that last for 2 min for a total of 8 h of data. These data are collected at the same time from 12 subjects performing a set of actions of interest randomly among arbitrary actions of non-interest in a continuous manner. This public domain dataset will be of benefit to researches conducting human action recognition in a more realistic setting of continuous action streams.

## Figures and Tables

**Table 1:** Actions of interest for the smart TV gestures application in the Continuous Multimodal Human Action Dataset (C-MHAD) dataset.

Action Label	Actions of Interest
1	swipe to left
2	swipe to right
3	hand wave
4	draw clockwise circle
5	draw counter-clockwise circle

**Table 2:** C-MHAD dataset.

	Smart TV Gestures	Transition Movements
Number of video clips	120	120
Length of each clip (minutes)	2	2
Number of subjects	12	12
Number of interested actions	5	7

**Table 3:** F1 scores of the detection stage in [31] applied to the C-MHAD dataset.

Sensing Modality	Smart TV Gestures F1 Score (%)	Transition Movements F1 Score (%)
Video only	77.8	81.1
Inertial only	60.3	60.9
Fusion of video and inertial	81.8	82.3

**Table 4:** Recognition accuracy of the recognition stage in [31] applied to the C-MHAD dataset for each action of interest—ND means no action detected.

	Actions of Interest	Video Only Accuracy (%)	Inertial Only Accuracy (%)	Fusion Accuracy (%)
**Smart TV Gestures**	swipe to left	95.0	82.4	100
	swipe to right	100	100	100
	hand wave	100	100	100
	draw clockwise circle	100	100	100
	draw counter-clockwise circle	100	100	100
**Transition Movements**	stand to sit	95.7	90.9	100
	sit to stand	85.7	85.7	90.0
	sit to lie	100	100	94.4
	lie to sit	86.7	90.9	93.3
	lie to stand	100	100	100
	stand to lie	100	ND	100
	stand to fall	100	100	100

**Table 5:** F1 scores of combined detection and recognition stages in [31] applied to the C-MHAD dataset.

Sensing Modality	Smart TV Gestures F1 Score (%)	Transition Movements F1 Score (%)
Video only	77.3	75.7
Inertial only	57.3	56.4
Fusion of video and inertial	81.8	78.8
